# Pediatric B-Cell Acute Lymphoblastic Leukemia: Comprehensive Genomic Characterization Including SNP-Array and Analysis of Relapse Risk

**DOI:** 10.3390/cancers18162633

**Published:** 2026-08-14

**Authors:** Concepción Prats-Martín, Laura Pérez Ortega, Águeda Molinos Quintana, Jordi Ribera, Beatriz Chiclana Rodríguez, Teresa Caballero-Velázquez, Estrella Carrillo Cruz, Henry Antonio Andrade-Ruiz, María Paz Garrastazul Sánchez, María Dolores Madrigal Toscano, María Solé Rodríguez, Marina Gómez Rosa, José Antonio Pérez-Simón, Rosario M. Morales-Camacho

**Affiliations:** 1 Department of Hematology, Hospital Universitario Virgen del Rocío, Instituto de Biomedicina de Sevilla (IBIS/CSIC/CIBERONC), Universidad de Sevilla, Avda. Manuel Siurot sn., CP 41013 Sevilla, Spain; laura.perez.ortega.sspa@juntadeandalucia.es (L.P.O.); rosario.morales.sspa@juntadeandalucia.es (R.M.M.-C.); 2 Josep Carreras Leukaemia Research Institute (IJC), ICO-Hospital Germans Trias i Pujol, Universitat Autònoma de Barcelona, 08916 Badalona, Spain; 3 Methodological and Statistical Support Unit, Fundación para la Gestión de la Investigación en Salud de Sevilla (FISEVI), 41013 Sevilla, Spain; 4 Department of Hematology, Hospital Universitario Puerta del Mar, 11009 Cádiz, Spain; 5 Department of Hematology, Hospital Universitario Virgen Macarena, 41009 Sevilla, Spain; 6 Department of Hematology, Hospital Universitario Juan Ramón Jiménez, 21005 Huelva, Spain; 7 UGC Hematología y Hemoterapia, Hospital Universitario Virgen de Valme, 41014 Sevilla, Spain

**Keywords:** B-cell acute lymphoblastic leukemia, SNP-array, relapse, pediatric, measurable residual disease

## Abstract

Background: Accurate identification of pediatric B-cell acute lymphoblastic leukemia (B-ALL) patients at increased risk of relapse remains a major clinical challenge, as relapse occurs in 10–20% of cases, including patients initially classified as low- or intermediate-risk. This study aimed to identify clinical, genomic, and measurable residual disease (MRD) related predictors of relapse in pediatric B-ALL. Methods: 51 pediatric patients with B-ALL were included and followed for a median of 30.5 months (IQR, 16–45.5). Patients were stratified according to relapse status. At diagnosis, all cases underwent comprehensive genomic characterization based on the 2022 WHO and ICC classifications, including SNP-array analysis to identify copy number alterations (CNA) involving recurrent B-ALL genes (*IKZF1*, *CDKN2A/B*, *PAX5*, *ETV6*, *BTG1*, *EBF1*, *ERG*, *RB1*, and *PAR1*) and to determine *IKZF1*^plus^ status. Clinical variables, including white blood cell count, cytogenetic risk, and MRD assessed by flow cytometry at day 15, day 33, and at the end of induction, were analyzed. Kaplan–Meier and Firth-penalized Cox regression analyses were performed to identify independent predictors of relapse. Results: 86.3% of patients were classified according to the 2022 WHO/ICC classifications, with high hyperdiploidy being the most frequent subtype. During follow-up, 11 patients relapsed. Relapse was significantly associated with high cytogenetic risk (*p* = 0.007) and showed a trend toward association with an adverse CNA profile (*p* = 0.075). Patients with >25% bone marrow blasts at day 15 (*p* < 0.001) and those with positive MRD at the end of induction (*p* = 0.017) had a significantly higher risk of relapse. In multivariable analysis, high genetic risk and positive end-of-induction MRD remained independent predictors of relapse, with hazard ratios (HRs) of 9.31 (95% CI, 1.55–56.1), *p* = 0.010, and 10.9 (95% CI, 2.39–49.9), *p* = 0.002, respectively. Conclusions: An integrated diagnostic strategy including SNP-array provides a high diagnostic yield. High-risk cytogenetic abnormalities and positive end-of-induction MRD are independent predictors of relapse in pediatric B-ALL. Their combined assessment at diagnosis and early treatment may improve risk stratification and may support personalized therapeutic approaches.

## Simple Summary

Despite the high cure rates achieved in pediatric B-cell acute lymphoblastic leukemia (B-ALL), relapse remains the leading cause of treatment failure and may occur even in children initially classified as low or intermediate risk. In this study, we used a Single Nucleotide Polymorphism array (SNP-array) as part of the diagnostic evaluation to perform comprehensive genomic profiling at diagnosis, enabling accurate classification according to the 2022 WHO and ICC criteria and the detection of clinically relevant copy number alterations. These genetic profiles were combined with measurable residual disease assessment by flow cytometry during treatment in order to assess their association with the risk of relapse. We found that high-risk genetic abnormalities identified at diagnosis and positive measurable residual disease at the end of induction were independent predictors of relapse. Our findings highlight the potential value of integrated genomic profiling including SNP-array, together with early treatment response assessment, for improving relapse risk prediction and personalizing treatment in pediatric B-ALL.

## 1. Introduction

Acute lymphoblastic leukemia (ALL) represents the single most frequently diagnosed form of cancer among children younger than 15 years. Registry-based epidemiological data from Spain and other European countries estimate the annual incidence at roughly 30–35 cases per million children [1,2]. B-cell precursor acute lymphoblastic leukemia (B-ALL) accounts for an estimated 85–90% of all pediatric ALL diagnoses [3]. This high incidence translates into ALL being the deadliest form of cancer among children [4].

Overall, children with B-ALL respond favorably to treatment, with high cure rates. Nevertheless, approximately 10–20% of patients experience relapse, and the prognosis in this setting remains significantly poorer [5]. In this context, novel therapeutic approaches, such as immunotherapy and chimeric antigen receptor T-cell (CAR-T) therapy, have provided new treatment options for these patients.

Several risk factors for relapse have been described in the literature and are currently used for prognostic stratification at diagnosis and for treatment allocation. These include patient-related factors, such as age, as well as disease-related characteristics, including leukocyte count, extramedullary involvement, and, most importantly, genetic alterations. Beyond these baseline features, response to therapy represents an independent determinant of outcome, particularly the persistence of measurable residual disease (MRD) after induction therapy [6]. A comprehensive characterization of B-ALL at diagnosis is therefore essential, as it largely determines clinical outcome. In particular, the identification of genetic alterations is critical not only for accurate disease classification according to the current 2022 World Health Organization (WHO) Classification of Haematolymphoid Tumours and the International Consensus Classification (ICC) [7,8], but also for risk stratification and the identification of potential therapeutic targets.

In recent years, the number of genetic alterations classically identified by conventional cytogenetics and fluorescence in situ hybridization (FISH) has grown exponentially, and novel genetic alterations have been identified that require alternative techniques for their detection. Single Nucleotide Polymorphism array (SNP-array) detects loss of heterozygosity (LOH) of genes or entire chromosomes, enabling the diagnosis of a particularly poor-prognosis subgroup, namely hypodiploidies. It also detects copy number alterations (CNAs) that have demonstrated prognostic significance, such as the *IKZF1*^plus^ [9]. Other subgroups, including *PAX5* amplification and intrachromosomal amplification of chromosome 21 (iAMP21), can likewise be identified using this technique. Furthermore, catastrophic genomic events such as chromoanagenesis can also be inferred from SNP-array data. The development of next-generation sequencing (NGS), including both DNA and RNA sequencing (RNA-seq), which detects point mutations and gene fusions, among others, complements the diagnosis and is being increasingly incorporated in routine diagnosis.

Another key assay in the study of B-ALL is flow cytometry, which, in addition to establishing B-cell lineage and maturation stage, can provide guidance toward possible genetic subtypes. For example, DUX4 rearrangements, which are difficult to detect by genetic techniques, can be inferred from the expression of CD371 and CD2 [10]. Other highly informative markers include the overexpression of the cytokine receptor *CRLF2*, which has a sensitivity of nearly 100% in the detection of Ph-like B-ALL with JAK-STAT pathway activation (primarily CRLF2::IGH and CRLF2::P2RY8 rearrangements, as well as CRLF2 point mutations) [11]. Furthermore, when NG2 expression is detected, a KMT2A rearrangement should be suspected. Flow cytometry also constitutes an essential component of the dynamic risk stratification of B-ALL during the early phases of treatment [12], with the persistence of MRD representing a critical factor in treatment intensification decisions within currently applicable pediatric chemotherapy protocols [6].

This study focuses on the diagnostic approach to pediatric B-ALL through the integration of cytometric and genetic techniques—particularly SNP-array—in a real-world cohort of 51 children. In addition, we performed a detailed analysis of relapses with the aim of identifying potential contributing factors to their understanding.

## 2. Materials and Methods

### 2.1. Patients and Treatment

The study cohort included 51 pediatric patients (aged ≤14 years) with B-ALL consecutively diagnosed between July 2021 and July 2025 across five hospital areas in Western Andalusia. Cytogenetic and molecular analyses were centrally performed at Virgen del Rocío University Hospital (Seville, Spain). Diagnostic studies were performed at diagnosis using bone marrow samples or, in selected cases, peripheral blood samples collected prior to treatment initiation. Diagnosis was based on the analysis of cytomorphology, flow cytometry, conventional cytogenetics, FISH, real-time polymerase chain reaction (PCR), SNP-array, and NGS. In three selected cases, the diagnostic workup was further complemented by RNA-seq. Cases were classified according to the 2022 WHO and ICC classifications [7,8]. In patients who relapsed with ≥20% blasts, genetic and molecular studies were repeated at relapse.

Regarding treatment, 50 of the 51 patients received chemotherapy. One patient died before treatment initiation and was therefore included only in the demographic and diagnostic classification analyses. Most patients (44/50, 88%) were treated in accordance with the SEHOP-PETHEMA 2013 protocol [13]. Among them, 4/44 (9.1%) were classified as low risk, 31/44 (70.5%) as intermediate risk, and 9/44 (20.5%) as high risk according to protocol-defined criteria. Two infants (2/50, 4%) were treated according to the Interfant-06 protocol and were classified as low risk according to protocol-defined criteria (*KMT2A* germline) [14]. Five patients (5/50, 10%) diagnosed after April 2025 received treatment in accordance with the pilot phase of the ALLTogether1 protocol [15]. High-risk patients and those who achieved a second complete remission after relapse or disease progression underwent consolidation with allogeneic hematopoietic stem cell transplantation (HSCT), which was performed in 8 patients. During follow-up, 11 relapses were documented. Relapse treatment included salvage chemotherapy in 5/11 patients, blinatumomab in 2/11 patients, inotuzumab in 5/11 patients, and CAR-T cell therapy in 7/11 patients.

### 2.2. Flow Cytometry Analysis

For diagnostic purposes, the Acute Leukemia Orientation Tube (ALOT) screening tube and the standardized and validated B-ALL extension tubes from the EuroFlow consortium were used [16]. Additionally, from 2023 onwards, a custom-designed tube was included, incorporating CD371-PE and CRLF2-APC, along with CD19-PE-Cy7, CD34-PerCP-Cy5.5, CD10-APC-H7, CD20-V450, and CD45-V500 for blast identification.

MRD was evaluated by next-generation flow cytometry, applying the EuroFlow consortium’s standardized sample processing, staining, and antibody panel procedures across two eight-color tubes [17]. Both tubes shared a common backbone of CD20-V450, CD45-V500, CD81-FITC, CD34-PerCP-Cy5.5, CD19-PE-Cy7, CD10-APC, and CD38-APC-H7, differing only in the PE channel, where either CD66c/CD123 or CD73/CD304 was incorporated. Following the standardized bulk lysis protocol and standardized instrument settings, this approach enabled detection of MRD in B-ALL samples at a sensitivity threshold of 10^−5^, provided that a minimum of 4 × 10^6^ cells were acquired per tube.

The limit of detection for MRD was set at 20 events and the limit of quantification at 50 events. In the increasingly common setting of MRD monitoring following anti-CD19 immunotherapy, the EuroFlow consortium has demonstrated that the use of EuroFlow MRD panels remains valid when applying CD19-independent analysis strategies [18]. MRD was assessed according to the protocol used at approximately days 15, 33, and the end of induction (day 70/78). Any result above the limit of detection was considered MRD-positive for the purpose of relapse risk analysis at day 33 and at the end of induction. Positivization of MRD was also considered as a relapse in subsequent assessments if the preceding result had been negative. At the day 15 assessment, two groups were defined based on the presence of 0–25% or >25% blasts by flow cytometry.

### 2.3. Genetic and Molecular Analysis

At diagnosis, all samples were evaluated by conventional cytogenetics, FISH, and real-time PCR. Cytogenetic analysis was performed by G-banding on 24-h cultures without mitogen stimulation, according to the laboratory’s standard procedures. The results were reported in accordance with the recommendations of the International System for Human Cytogenomic Nomenclature (ISCN) 2024 [19]. FISH was performed using the following commercial probes: *BCR::ABL1*, *ETV6::RUNX1*, *TCF3::PBX1*, *KMT2A*, *ABL1*, *ABL2*, *PDGFRB*, *CSF1R*, *JAK2*, *MYC* and *IGH* (Vysis, Abbott Laboratories, Chicago, IL, USA) and *TCF3::HLF* (Cytocell, Ltd., Cambridge, UK). Real-time PCR was additionally performed for the following four rearrangements: *BCR::ABL1*, *ETV6::RUNX1*, *TCF3::PBX1* and *KMT2A::AFF1*. Three cytogenetic subgroups were considered: good risk (t(12;21)/*ETV6*::*RUNX1* and high hyperdiploidy), poor risk [11q23/*KMT2A* rearrangement, t(9;22)/*BCR*::*ABL1*, iAMP21, low hypodiploidy or haploidy (<40 chromosomes) and t(17;19)/*TCF3*::*HLF*] and intermediate risk [t(1;19)/*TCF3*::*PBX1* and B-other] [20].

Genomic DNA for SNP-array and NGS studies was isolated from bone marrow or peripheral blood mononuclear cells using a DNA kit from Qiagen (Venlo, The Netherlands) according to the manufacturer’s instructions from 2018 to 2022 and by using automated DNA extraction (Chemagic 360 instrument, Revity, Waltham, MA, USA) from 2023 to 2025. DNA samples were quantified by the fluorometric Qubit assay.

SNP-array analysis was carried out in cases without recurrent genetic alterations until May 2024 and onwards in all cases. SNP-array was performed at diagnosis for 41/51 B-ALL patients and was repeated in 2 of 11 patients at relapse. A total of 2 out of 43 samples were analyzed using the CytoScan 750K platform (750,000 markers, including 200,000 SNPs; Thermo Fisher Scientific, Waltham, MA, USA), while the remaining samples were processed using the CytoScan HD platform (2,670,000 markers, including 750,000 SNPs; Thermo Fisher Scientific, Waltham, MA, USA). SNP-array data were analyzed using Chromosome Analysis Suite software version 4.5 (ChAS; Thermo Fisher Scientific). All data were aligned to the hg38 human genome reference assembly. CNVs > 5 Mb and microdeletions involving the *IKZF1*, *CDKN2A/B, PAX5, ETV6, BTG1*, *EBF1*, *ERG,* and *RB1* genes and the pseudoautosomal region 1 (*PAR1*), regardless of size, were ascertained. Additionally, only copy-neutral loss of heterozygosity (CN-LOH) regions on autosomes exceeding 10 Mb and involving telomeric regions were reported. The analysis followed the recommendations of the American College of Medical Genetics and Genomics (ACMG) Technical Standard for chromosomal microarray analysis and interpreting CNAs in neoplastic disorders [21]. Regarding CNAs, patients were defined as *IKZF1*^plus^ positive if an *IKZF1* deletion co-occurred with deletions of *CDKN2A*, *CDKN2B* (homozygous deletion only), *PAX5,* or the pseudoautosomal region 1 (*PAR1*) [22].

CytoScan 750K platform (750,000 markers, including 200,000 SNPs; Thermo Fisher Scientific, Waltham, MA, USA) was used in 2 of 43 samples, whereas the CytoScan HD platform (2,670,000 markers, of which 750,000 are SNPs; Thermo Fisher Scientific, Waltham, MA, USA) was applied to the remaining samples. Data analysis was performed with Chromosome Analysis Suite software, version 4.5 (ChAS; Thermo Fisher Scientific), using the hg38 genome build as reference. We considered CNVs larger than 5 Mb, together with microdeletions affecting *IKZF1*, *CDKN2A/B*, *PAX5*, *ETV6*, *BTG1*, *EBF1*, *ERG*, or *RB1*, as well as any deletion—irrespective of size—within the pseudoautosomal region 1 (*PAR1*). Copy-neutral loss of heterozygosity (CN-LOH) was reported only when spanning more than 10 Mb of autosomal, telomere-including regions. All interpretations adhered to the American College of Medical Genetics and Genomics (ACMG) Technical Standard for chromosomal microarray analysis and interpreting CNAs in neoplastic disorders [21]. A case was classified as *IKZF1*^plus^ when an *IKZF1* deletion co-occurred with at least one additional deletion in *CDKN2A*, *CDKN2B* (homozygous loss only), *PAX5*, or *PAR1* [22].

Patients with *ERG* deletions were excluded from this category, as *ERG* deletion has been shown to mitigate the adverse prognostic impact of the *IKZF1*^plus^ profile [23]. The non-*IKZF1*^plus^ group included all other patients, regardless of *IKZF1* status (mutated or wild-type). Additionally, the United Kingdom CNA (UKALL-CNV) classifier was used as a prognostic tool. Although the original classifier defines three risk groups (good risk, intermediate risk, and poor risk), for the purposes of this study, patients were grouped into two categories: “good” risk and “not good” risk, with the latter encompassing both the intermediate risk and poor risk groups [9].

NGS was performed in 24 patients at diagnosis and 6 patients at relapse. For NGS analyses, a total amount of 10 ng was employed to perform a PCR-based targeted sequencing of a panel that included *IKAROS*, *ETV6, FLT3*, *RAS* signaling pathway, and *TP53* (Oncomine Myeloid Research panel from 2018 to 2024 and Oncomine Myeloid Assay GX from 2024 to 2025).

Samples were sequenced on either the Ion Torrent S5 or the Genexus System (Life Technologies, Carlsbad, CA, USA). Sequencing raw data were processed with the corresponding analysis pipeline, using Ion Torrent Suite Software v5.20 or Genexus Software (Oncomine Myeloid DNA Assay Gx5 v.5.04) for read alignment and variant detection. Variant annotation and clinical reporting were generated with Ion Reporter or Genexus Software. The clinical significance of the detected variants was assessed according to the Association for Molecular Pathology, American Society of Clinical Oncology, and College of American Pathologists guidelines for sequence variant interpretation [24]. RNA-seq was additionally performed in three patients with the Oncomine Childhood Cancer Research Assay.

### 2.4. Statistical Analysis

Statistical analyses were carried out with R software, version 4.4.3. Categorical data are presented as numbers and percentages. Quantitative variables were summarized according to their distribution. Normality was examined using the Shapiro–Wilk test; as the continuous variables did not follow a normal distribution, they are described as medians with interquartile ranges (IQR, 25th–75th percentiles). Comparisons between patients with and without relapse were performed using Pearson’s chi-square test or Fisher’s exact test for categorical variables, as appropriate based on expected cell counts. Continuous variables were compared using the Mann–Whitney U test.

The primary endpoint was time to relapse. Relapse-free survival was assessed using Kaplan–Meier estimates, and differences between groups were explored with the log-rank test. Because this endpoint included time-to-event information, Firth-penalized Cox regression was selected as the main inferential method. This approach was used to reduce potential bias and improve the stability of estimates in the setting of a limited sample size, few relapse events, and sparse categories. Effect estimates are reported as hazard ratios (HRs) with 95% confidence intervals. For Cox models, the proportional hazards assumption was checked using Schoenfeld residuals and graphical evaluation. The possible influence of very small strata on model estimates was also considered.

As a complementary analysis, Firth-penalized logistic regression models were fitted to evaluate the consistency of associations when relapse was treated as a binary outcome. These models were reported using odds ratios (OR) with 95% confidence intervals and were interpreted as complementary to the primary time-to-relapse analysis (Supplementary Material).

The main multivariate model was pre-specified to include genetic risk and end-of-induction MRD. This model was considered the primary approach because it preserved the largest number of available observations and combined two complementary prognostic dimensions: the baseline genetic biology of leukemia and the treatment response measured by MRD.

When missing data were present, a model-specific complete-case approach was applied so that each analysis used the maximum number of patients with available data for the variables included. A two-sided *p* value < 0.05 was considered statistically significant.

## 3. Results

### 3.1. Baseline Characteristics and Differences According to Relapse Status

A total of 51 children with B-ALL were included in the analyses of diagnostic characteristics. 50 patients received treatment, as one patient died before treatment initiation. Consequently, only the 50 treated patients were included in the analyses comparing patients with and without relapse (Table 1). During follow-up, 11 of the 50 treated patients (22%) experienced relapse.

The cohort comprised 28 males (55%) and 23 females (45%), with a median age at diagnosis of 3 years (IQR, 2–6; range, 0.8–14 years). No significant differences were observed between patients with and without relapse regarding sex distribution, age at diagnosis, or baseline leukocyte count, hemoglobin level, and platelet count (Table 1). Flow cytometric analysis revealed a common B-ALL immunophenotype in 39 cases (76.5%) and a pre-B ALL immunophenotype in 12 cases (23.5%).

According to the 2022 ICC classification, 44 of the 51 cases (86.3%) were classified as B-ALL with recurrent genetic abnormalities, whereas the remaining 7 cases (13.7%) were classified as B-ALL, not otherwise specified (NOS). The most frequent genetic subtypes were high hyperdiploid B-ALL, comprising 23 cases (45.1%), and B-ALL with t(12;21)(p13.2;q22.1)/*ETV6*::*RUNX1*, comprising 8 cases (15.7%). Less frequent subtypes included two cases (3.9%) of hypodiploid (one B-ALL being low hypodiploid and one B-ALL being near haploid), two cases (3.9%) of B-ALL with t(1;19)(q23;p13.3)/*TCF3*::*PBX1*, two cases (3.9%) of B-ALL *BCR*::*ABL1*-like, JAK-STAT activated (*CRLF2*::*P2RY8* rearrangement), two cases (3.9%) of B-ALL with iAMP21, and two cases (3.9%) of the provisional entity B-ALL, *PAX5*-altered (PAX5alt), including one case with intragenic amplification of exons 2 to 5 and one with a *PAX5*::*NOL4L* rearrangement. Finally, single cases (2.0% each) of B-ALL with t(9;22)(q34.1;q11.2)/*BCR*::*ABL1*; B-ALL with t(9;11)(p21.3;q23)/*KMT2A*::*MLLT3*; and B-ALL with *DUX4*-rearrangement were identified (Table 1). According to the 2022 WHO classification, differences in diagnostic categorization were minimal, as *DUX4*-rearranged B-ALL and PAX5alt B-ALL were both included within the category of B-ALL with other defined genetic abnormalities.

Of these 44 classified cases, 12 (27.3%) were diagnosed using conventional cytogenetic techniques, mainly based on recurrent gene rearrangements (all confirmed by FISH). Seventeen patients (38.6%) were classified by SNP array, including 8 cases of high hyperdiploidy (6 with failed cell culture and 2 with a normal karyotype). In the remaining 15 cases of high hyperdiploidy (34.1%), SNP array refined the diagnosis by accurately defining the pattern of chromosomal gains and excluding masked hypodiploidy. Overall, SNP array either established or refined the cytogenetic classification in 72.7% of classified cases, allowing a total of 86.3% of pediatric B-ALL cases to be assigned to an ICC 2022 genetic subtype.

According to cytogenetic risk stratification, 29 of the 51 cases (57%) were classified as having good risk, 17 (33%) as intermediate risk, and 5 (10%) as poor risk. The relapse rates were 2/28 (7.1%) in the good-risk group, 6/17 (35.3%) in the intermediate-risk group, and 3/5 (60.0%) in the poor-risk group, with significant differences among the groups (*p* = 0.007).

The most frequent CNAs detected at diagnosis (available in 41 patients) were deletions of *CDKN2A* (13/41, 31.7%), *CDKN2B* (12/41, 29.3%), *IKZF1* (10/41, 24.4%), *PAX5* (9/41, 22%), *ETV6* (8/41, 19.5%), *PAR1* (2/41, 4.9%), *RB1* (2/41, 4.9%), *EBF1* (2/41, 4.9%), and *BTG1* (1/41, 2.4%). The frequency of the *IKZF1*^plus^ profile was 8/41 (19.5%).

Among the 41 cases analyzed, 23 (56%) were classified as having a “good” risk CNA profile and 18 (44%) as having a “not good” risk CNA profile. A non-significant trend toward a higher relapse rate was observed in the “not good” risk CNA profile (7/18, 38.9%) compared with the “good” risk group (3/23, 13.0%) (*p* = 0.075). No significant association was observed between *IKZF1*^plus^ status and relapse (*p* = 0.4).

Target NGS analysis was performed in 24 cases. Nine patients (37.5%) harbored a single mutation, two (8.3%) harbored two mutations, and three (12.5%) harbored more than two mutations; no mutations were detected in 10 patients (41.7%). The most frequently mutated genes were those involved in the RAS signaling pathway: *NRAS* (n = 7), *KRAS* (n = 5), *PTPN11* (n = 4), and *CBL* (n = 1). Other mutated genes included *FLT3* (n = 3), *IKZF1* (n = 2), and *ETV6* (n = 1). Mutations affecting the RAS signaling pathway were more frequent in hyperdiploid cases (9/14 cases with available NGS data, 64.3%) than in non-hyperdiploid cases (2/10, 20.0%) (*p* = 0.017).

Regarding treatment response at day 15, one patient was excluded because of missing data (evaluable n = 49). Forty-four patients had a bone marrow blast percentage ≤ 25%, whereas five had >25%, which was strongly associated with relapse (*p* < 0.001). At day 33, 31 patients (62%) achieved complete remission (CR) with negative MRD, whereas 19 (38%) had detectable MRD, with a trend towards more relapses in the detectable MRD group (*p* = 0.078). After induction, 44 patients (88%) achieved CR with negative MRD and six (12%) achieved CR with positive MRD. The end of induction MRD status was significantly associated with relapse: 4/6 patients (66.7%) with positive MRD relapsed compared with 7/44 (15.9%) with negative MRD (*p* = 0.017) (Table 1).

During follow-up, 11 patients experienced relapse, of whom four subsequently died. At a median follow-up of 30.5 months (IQR, 16.0–45.5 months), overall survival was 90.2% (46/51).

### 3.2. Risk Factors for Relapse

Univariable analysis identified cytogenetic risk, >25% blasts at day 15 assessment, and end-of-induction MRD as significant risk factors for relapse.

#### 3.2.1. Genetic and Molecular Alterations as Risk Factors for Relapse

**Figure 1:**
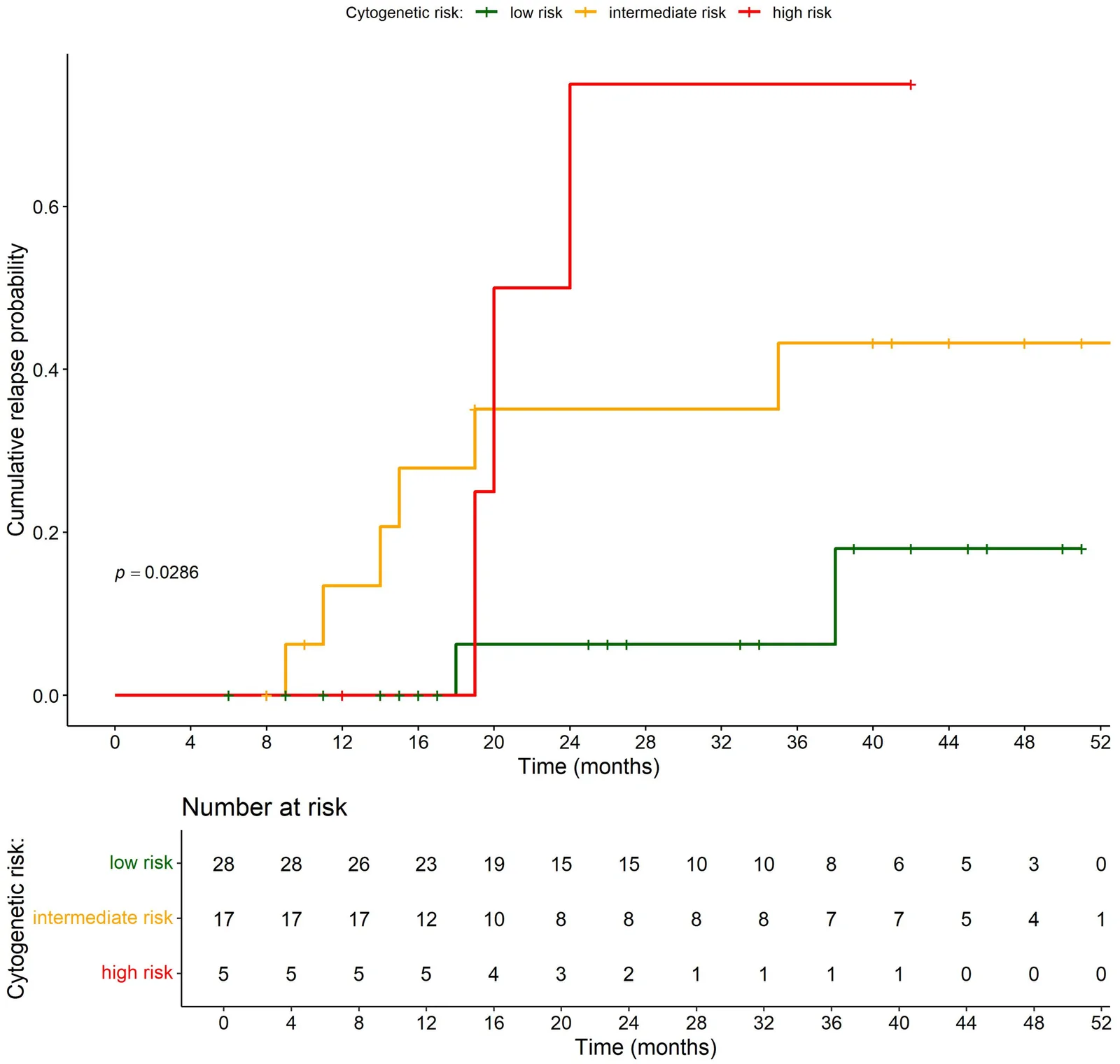
Kaplan–Meier analysis for correlation between cumulative relapse probability and cytogenetic risk.

The cumulative probability of relapse, estimated using the Kaplan–Meier method, differed significantly according to cytogenetic risk (Figure 1). In univariable Firth-penalized Cox regression, cytogenetic risk was associated with time to relapse (Table 2). Compared with the good-risk group, intermediate risk showed HR 4.09 (95% CI 0.86–19.4; *p* = 0.044) and poor risk showed HR 7.21 (95% CI 1.27–40.9; *p* = 0.020).

**Figure 2:**
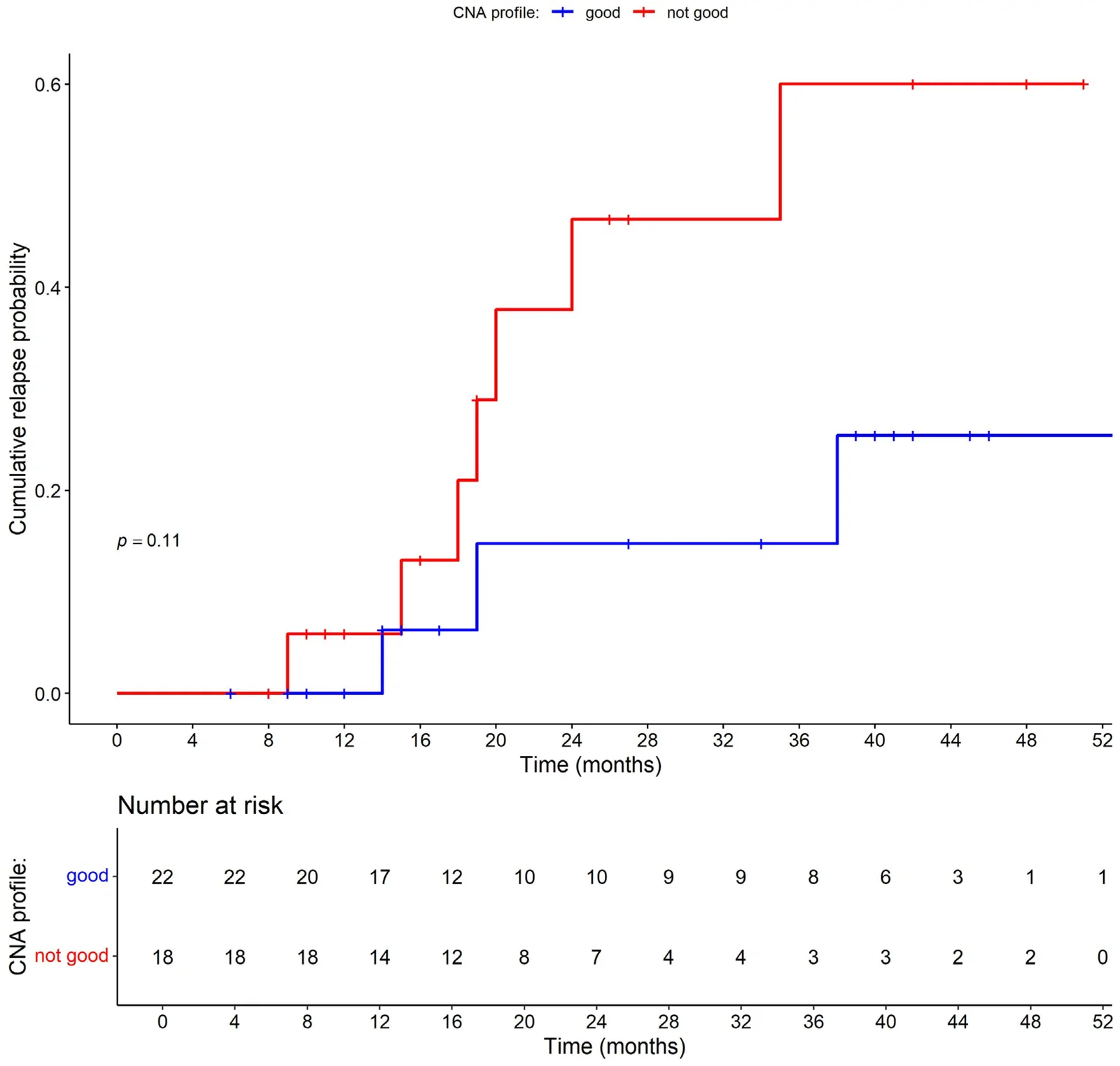
Kaplan–Meier analysis for correlation between cumulative relapse probability and CNA profile.

The cumulative probability of relapse, estimated using the Kaplan–Meier method according to CNA profile, showed a trend toward worse relapse-free survival in the “not good” group compared with the “good” group, although this did not reach statistical significance (Figure 2). Likewise, a non-significant trend toward a higher risk of relapse was observed for the “not good” CNA profile (HR 2.94; 95% CI 0.77–11.3; *p* = 0.085) in univariable Firth-penalized Cox regression (Table 2).

#### 3.2.2. MRD as a Risk Factor for Relapse

**Figure 3:**
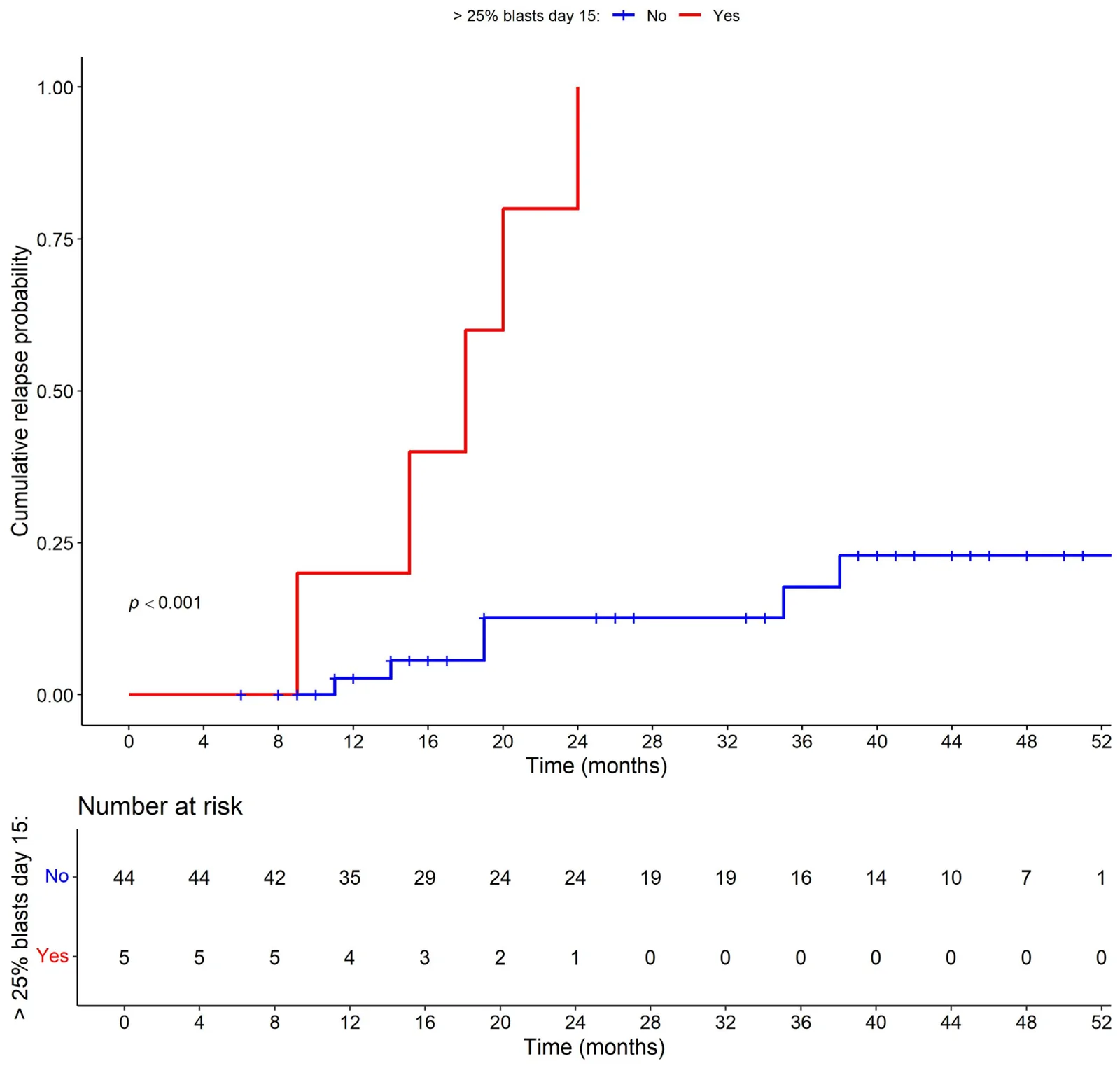
Kaplan–Meier analysis for correlation between cumulative relapse probability and >25% blasts on day 15.

In the Kaplan–Meier analysis of time to relapse according to the presence or absence of >25% blasts at day 15, one observation was excluded due to missing data (evaluable n = 49). A higher incidence of early relapse in the group with >25% blasts at day 15 was observed (Figure 3). >25% blasts at day 15 also showed a strong association with relapse (HR 13.4; 95% CI 3.53–50.8; *p* < 0.001) in the Firth-penalized Cox model.

**Figure 4:**
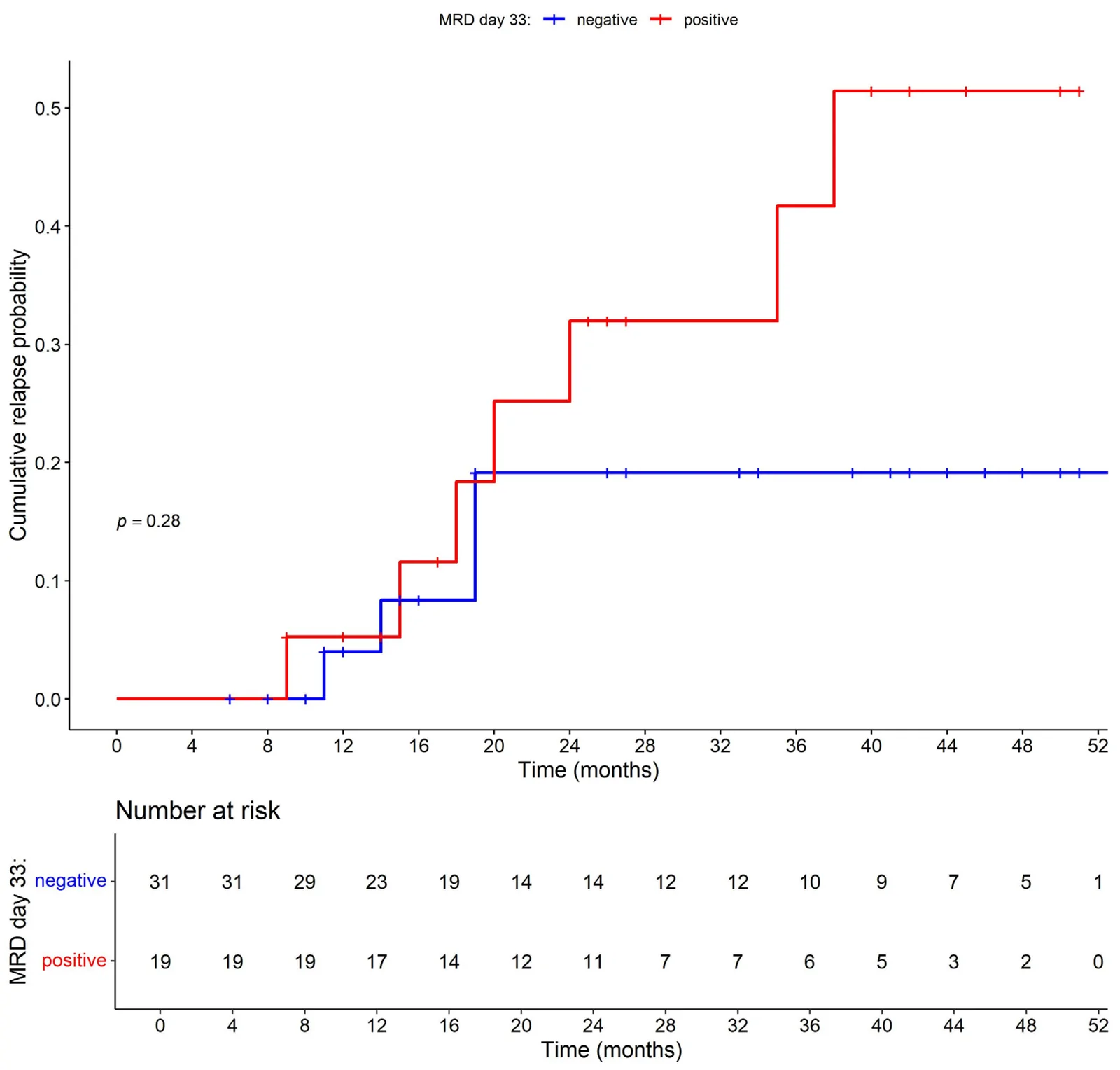
Kaplan–Meier analysis for correlation between cumulative relapse probability and MRD day 33.

The Kaplan–Meier analysis of time to relapse according to MRD status on day 33 did not show statistically significant differences (Figure 4). Consequently, MRD status on day 33 was not statistically significant (N = 50; 11 events; HR 2.32; 95% CI 0.68–7.87; *p* = 0.20) in the Cox model (Table 2).

**Figure 5:**
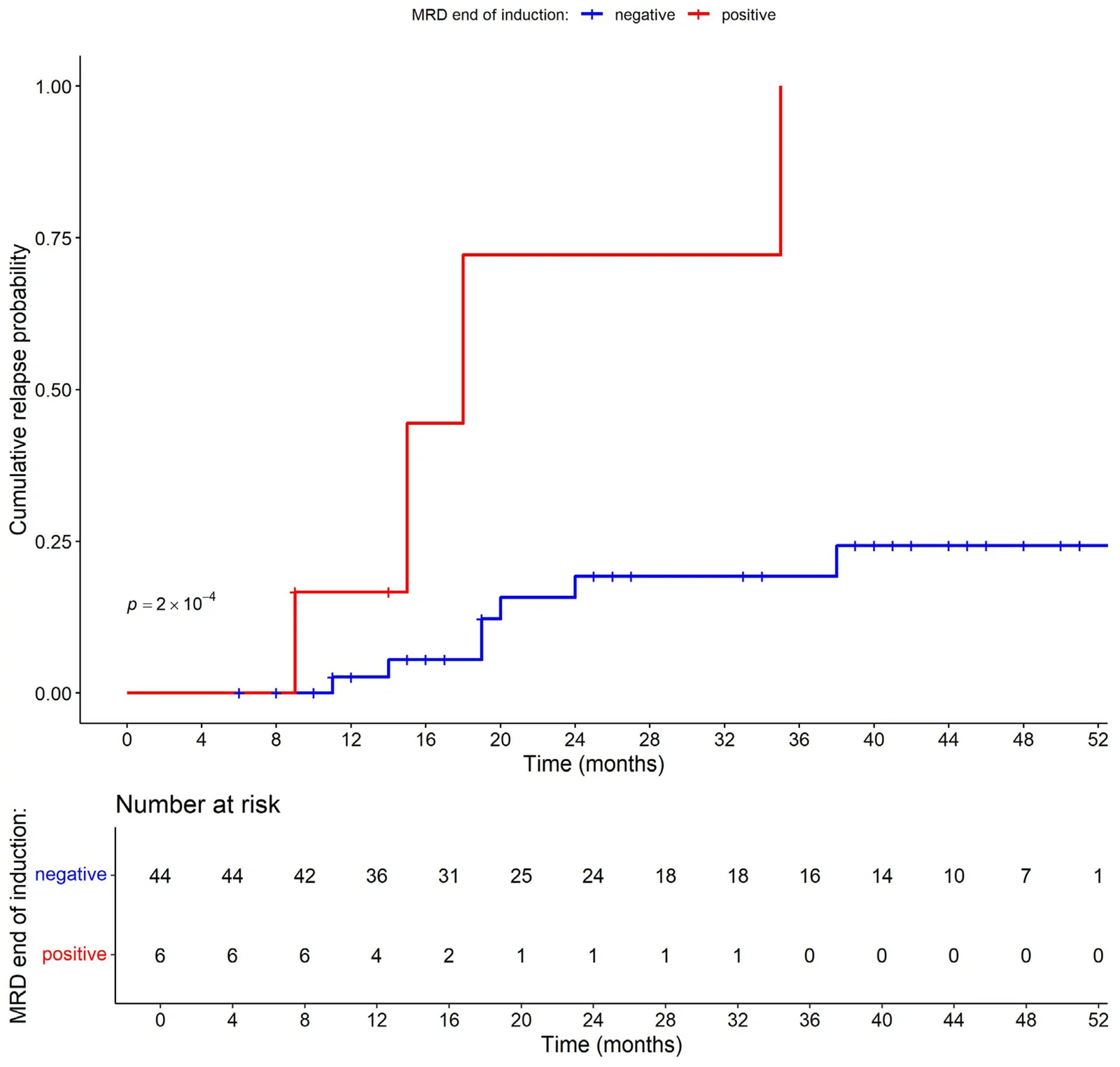
Kaplan–Meier analysis for correlation between cumulative relapse probability and MRD end of induction.

Finally, for end-of-induction MRD status, the Kaplan–Meier analysis showed significant differences in relapse-free survival (Figure 5). In the univariable Firth-penalized Cox model, MRD positivity at the end of induction was associated with a marked increase in the hazard of relapse compared with MRD negativity (HR 10.2; 95% CI 2.78–37.4; *p* = 0.001) (Table 2).

#### 3.2.3. Multivariable Analysis of Risk Factors for Relapse

In the primary multivariable model (Table 3), defined a priori and including genetic risk and end-of-induction MRD, MRD positivity was independently associated with higher relapse risk (HR 10.9; 95% CI 2.39–49.9; *p* = 0.002). Poor cytogenetic risk also remained independently associated with a higher risk of relapse as compared to the good-risk group (HR 9.31; 95% CI 1.55–56.1; *p* = 0.010), whereas intermediate risk showed a non-significant increase (HR 2.56; 95% CI 0.50–13.1; *p* = 0.3).

In the sensitivity analysis replacing end of induction MRD with >25% blasts at day 15, the “Yes” category was associated with higher relapse risk (HR 18.8; 95% CI 3.11–114; *p* = 0.001). However, this result should be interpreted cautiously because the exposed subgroup was very small, and all exposed patients relapsed. Therefore, attenuation of the cytogenetic risk effect in this model should not be interpreted as a lack of biological relevance, but rather as a consequence of sparse data and the strong relationship between poor early response and relapse (Supplementary Material).

The multivariable Cox–Firth analyses should be interpreted with caution because of the limited sample size, the small number of relapse events, and the presence of sparse categories, which resulted in wide confidence intervals and limited precision of the estimates. These analyses were exploratory and were not intended to develop or validate a clinical prediction model. Therefore, model performance measures such as discrimination, calibration, or internal validation were not performed, and the findings require confirmation in larger independent cohorts.

## 4. Discussion

We present a comprehensive analysis of 51 pediatric patients diagnosed with B-ALL, focusing on the genetic characteristics at diagnosis, treatment response, and the features observed at relapse in the 11 patients who experienced disease recurrence. Our aim was to identify factors potentially associated with relapse risk and to provide further insights into the biological and clinical heterogeneity of the disease. Regarding the diagnostic characteristics of the 51 cases, by applying an integrated diagnostic approach based on cytomorphology, flow cytometry, karyotyping/FISH, real-time PCR, SNP-array, and targeted NGS, we were able to assign a genetically defined subtype according to the current 2022 ICC and WHO classifications in 86.3% of patients. This substantially reduces the proportion of cases categorized as B-ALL NOS compared with previous classification systems based on conventional techniques (karyotyping, FISH, and PCR), in which approximately 30% of cases historically remained unclassified [25,26]. These results are comparable to the diagnostic yield reported with the additional use of optical genome mapping (OGM) (83%) [27]. They are also close to those reported in other series incorporating transcriptomic techniques (RNA-seq) in B-other cases, in which a genetic subtype can be assigned in 90–96% of patients [25,28]. High hyperdiploid B-ALL was the most common subtype (45.1%), followed by B-ALL with *ETV6*::*RUNX1* fusion (15.7%). The higher frequency of hyperdiploid cases compared with that reported in many international pediatric series (approximately 26–38%) [9,25,27,28,29] may be related to the relatively small size of our cohort. Nevertheless, this overrepresentation of high hyperdiploid cases did not substantially affect the overall distribution of cytogenetic risk categories, which was consistent with that reported in the literature [20], with a predominance of good-risk subtypes accounting for 57% of cases, whereas intermediate- and high-risk subtypes represented 33% and 9.8% of cases, respectively.

Regarding CNAs, the most commonly deleted genes were *CDKN2A*, *CDKN2B*, *IKZF1*, *PAX5*, and *ETV6*, in keeping with previous reports in pediatric B-ALL [9,29,30,31]. The frequency of the *IKZF1*^plus^ genotype, which identifies a subgroup of patients with an adverse prognostic profile and an increased risk of relapse [22,32], was 19.5% (8/41) in our cohort, slightly higher than that reported in most unselected pediatric series (approximately 6–15%) [9,22,29,31]. All *IKZF1*^plus^ cases occurred within the intermediate or poor cytogenetic risk categories. Among the eight patients with an *IKZF1*^plus^ profile, three experienced relapse and one subsequently died; however, these findings did not reach statistical significance.

NGS analysis was performed in 24 patients, and at least one mutation was detected in 58.3% of cases. Consistent with previous reports, alterations affecting the RAS signaling pathway, particularly *NRAS*, *KRAS*, and *PTPN11*, were the most common alterations [31,33,34]. A total of 22% of children in our cohort relapsed, which is consistent with the relapse incidence reported in the literature, estimated at around 20% using similar treatment regimens [35]. The overall survival of 90.2% observed in our cohort is consistent with the excellent outcomes expected with contemporary MRD-guided pediatric ALL treatment strategies, including the ALLTogether1 protocol, which was designed in the context of childhood ALL survival rates exceeding 90% [15]. Cytogenetic risk was identified as a risk factor for relapse, in concordance with multiple studies, confirming the validity in our setting of the prognostic stratification used in current treatment protocols, which are primarily based on this factor [36]. In line with this, the CNA profile, validated as a predictor of relapse-free survival in a large study of 3239 cases collected from 12 groups within the International BFM Study Group [9], approached statistical significance in our cohort, likely due to the limited sample size. Although the *IKZF1*^plus^ genotype was more frequent in relapses (30% vs. 16%), no statistically significant association was demonstrated in our series, possibly due to the limited number of cases. This genotype has been associated with a 2.2-fold increase in relapse risk in high-risk patients but a non-significant 1.5-fold increased risk in standard-risk patients in the NCI cohort, and other studies have shown that *IKZF1* deletions carry an adverse prognosis across all risk groups [37]. Furthermore, it has been described that the combination of MRD and *IKZF1* deletion allows for better risk stratification of adults with B-ALL, and that allogeneic transplant mitigates the poor prognosis of MRD-positive and/or *IKZF1*^plus^ subgroups [38,39]. Regarding response to chemotherapy, differences in relapse were found according to the end of induction MRD, which has been described as the time point with the greatest predictive value for relapse in both pediatric and adult populations [40]. However, it should be noted that the definition of MRD positivity used in our analysis (any detectable MRD above the limit of detection) differed from the threshold used for clinical risk stratification and treatment decisions in the SEHOP-PETHEMA 2013 protocol (≥0.01%) [41]. Therefore, although our findings suggest that even very low levels of MRD retain prognostic significance, this cutoff should be prospectively validated in larger pediatric cohorts before being considered for clinical implementation. Another response assessment time point that predicts relapse is the persistence of more than 25% blasts at day 15 in our cohort, which corresponds to slow responders. It has been reported in a pediatric cohort that slow response was associated with a 5-year event-free survival of 53.3% versus 83.9%, regardless of treatment intensification [42], although other groups did not find this difference [43].

A more detailed analysis of relapse cases stratified by genetic risk was performed (Table 4). Interestingly, in the good cytogenetic risk group, two patients relapsed, both showing high hyperdiploidy of 54 and 57 chromosomes, respectively. In the literature, high hyperdiploidy has been reported to account for up to 25% of all relapses [44], as it represents the most common cytogenetic subgroup in childhood ALL. In our series, these cases accounted for 2 of the 11 relapses (18.2%). The first child showed a poor initial response to chemotherapy, with >25% blasts on day 15 and persistent end-of-induction MRD, factors that were associated with an increased risk of relapse in this series and that are consistent with findings reported in larger high hyperdiploidy cohorts [45]. This highlights the importance of integrating genetic data with MRD assessment. Other factors that may have contributed to relapse included central nervous system (CNS) involvement, *CDKN2A/B* deletion, and *IKZF1* and *KRAS* mutations. In this regard, it is well established that high hyperdiploid cases may harbor RAS pathway and receptor tyrosine kinase mutations in up to 51% of cases, without clearly affecting prognosis [46]. In contrast, *IKZF1* mutations (including single nucleotide variants and small indel mutations) are very uncommon in pediatric B-ALL (2.5%) and have been associated with reduced glucocorticoid sensitivity and increased MRD on day 15 of induction [47]. The second case of high hyperdiploidy corresponds to a girl who had no apparent risk factors for relapse at presentation and received standard-risk chemotherapy with a good initial response. She harbored trisomy 5, which is associated with an increased risk of relapse according to the UKALL classification, which describes a high-hyperdiploid poor-risk profile. This study, based on trisomies involving chromosomes 17, 18, 5, and 20, identifies the presence of trisomy 5 or 20 as an adverse prognostic factor independent of MRD [45]. In addition, RNA-seq was performed in this patient, which ruled out underlying rearrangements associated with hyperdiploidy. Both children are currently alive and remain in complete remission with negative MRD.

**Figure 6:**
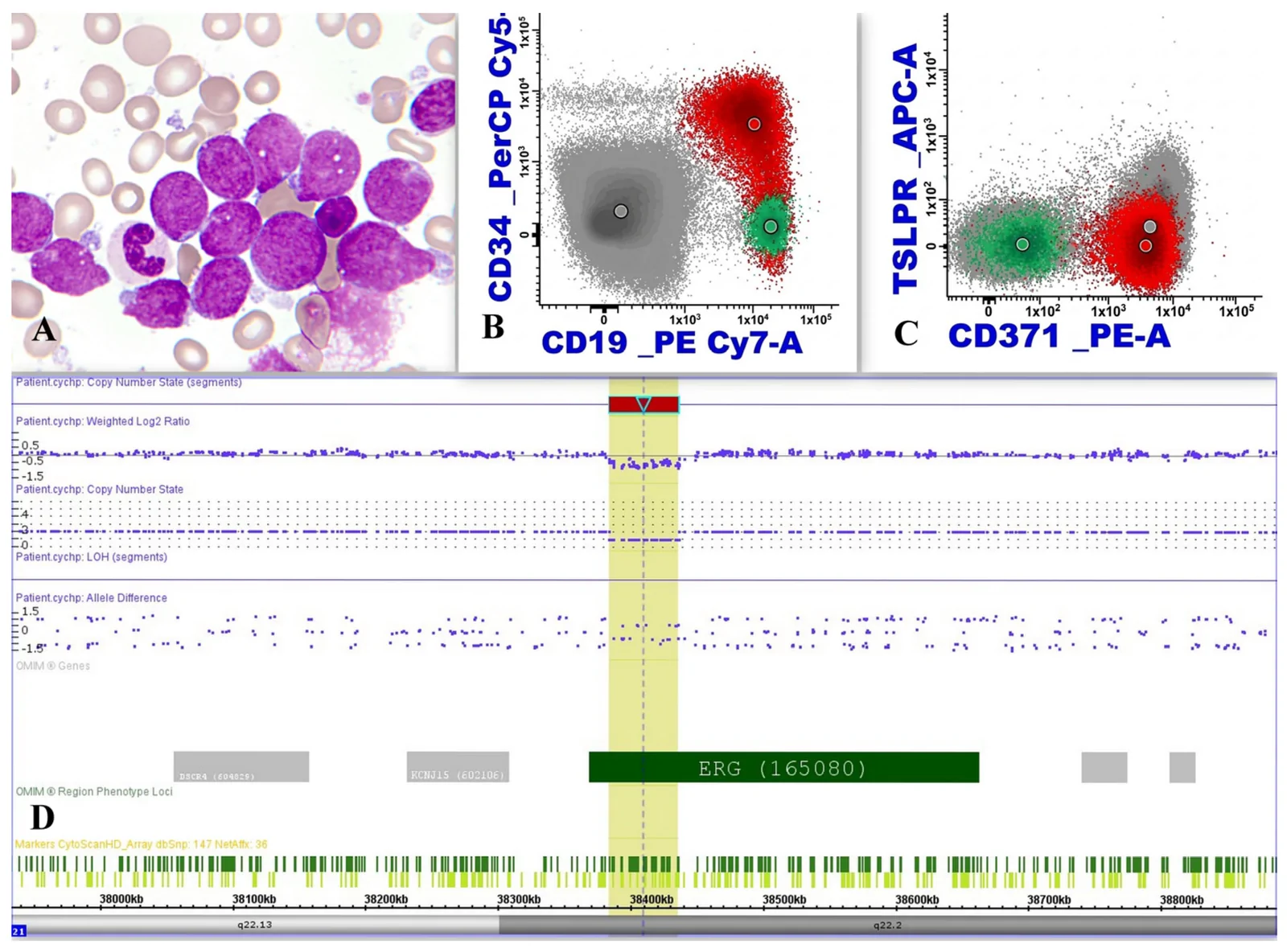
B-ALL with *IGH*::*DUX4* rearrangement. Bone marrow. (**A**) May–Grünwald–Giemsa stain, ×100. Cytology showing infiltration by lymphoid-appearing blasts. (**B**,**C**) Flow cytometry: in green, normal CD19+ B lymphocytes; in red, B lymphoid blasts expressing CD371. (**D**) SNP array showing intragenic deletion of the *ERG* gene.

The highest number of relapses occurred in the intermediate cytogenetic risk category, which included six children. The first child in this group harbored an *IGH*::*DUX4* rearrangement (Figure 6), which has been described as being associated with a favorable prognosis. This rearrangement was initially based on CD371 expression detected by flow cytometry together with an *ERG* intragenic deletion identified by SNP array, both considered surrogate markers of this rearrangement. Subsequently, the *IGH*::*DUX4* rearrangement was confirmed by RNA-seq with gene expression profiling and fusion analysis after the sample was submitted to the Spanish national SEHOP-PENCIL project (Personalised mEdiciNe for Cancer in chILdren in Spain). Among the features that could be related to relapse, the patient presented with older age (12 years), extramedullary involvement, cytogenetics showing trisomy 21 in a subclone, *CDKN2A/B* deletion, and a biallelic deletion of *TBL1XR1*, which has been associated with glucocorticoid resistance [48]. In addition, a point mutation in *IKZF1* was detected. The clinical course was unfavorable, and the patient died following a fourth relapse due to disease progression. The second case in this group was B-ALL, NOS with marked hypereosinophilia and an unusual karyotype with clones showing near-triploidy and tetraploidy, along with an isochromosome 17q (resulting in *TP53* deletion in one of the three chromosome 17 copies), without detectable *TP53* mutation and without evidence of a hypodiploid clone. In the study by Pui and colleagues, which included 2805 children with ALL, i(17q) was identified in 23 cases (0.8%), being considered a rare but recurrent abnormality in B-ALL that is frequently associated with high hyperdiploidy. In that study, it was associated with a high relapse rate and relatively short remission durations [49]. Other adverse prognostic factors at diagnosis included age (10 years) and an unfavorable CNA profile, with *CDKN2A/B* and *PAX5* deletions. In addition, during disease evolution, the patient showed a slow early response, with >25% blasts on day 15 and persistent end-of-induction MRD, both of which are associated with an increased risk of relapse. The patient died after a third medullary relapse due to infection and pulmonary hemorrhage.

The third case was an 11-month-old infant with B-ALL harboring a *PAX5* alteration with an in-frame *PAX5*::*NOL4L* gene fusion. The oncogenic effect is thought to derive primarily from functional disruption of *PAX5*, a gene that regulates B-cell differentiation [50]. Additional adverse prognostic factors at diagnosis that may have contributed to relapse included: age <1 year, hyperleucocytosis, and an unfavorable CNA profile classified as *IKZF1*^plus^. The patient also harbored a *KRAS* mutation, which has been described as a frequent cooperating alteration in this genetic subgroup. In addition, she showed >25% blasts on day 15 and positive end-of-induction MRD. She is currently in complete remission with negative MRD.

The fourth relapsed case in this group was a 22-month-old boy with *BCR*::*ABL1*-like B-ALL, with activation of the JAK–STAT pathway driven by a *CRLF2*::*P2RY8* rearrangement. At diagnosis, risk factors for relapse included hyperleucocytosis, and the CNA profile was classified as *IKZF1*^plus^. During disease evolution, the end-of-induction MRD remained positive. The combination of *CRLF2*::*P2RY8* rearrangement, *IKZF1*^plus^ profile, and positive end of induction MRD observed in this case is considered to underlie the poor prognosis in this category [51,52]. The patient died due to septic shock in the setting of active disease.

The fifth child was B-ALL, NOS. At diagnosis, no classical risk factors for relapse were identified. SNP-array analysis revealed only an 894 kb microdeletion involving a complete deletion of the *VPREB1* gene. This gene plays a central role in B-cell development as part of the pre-B cell receptor, and its loss is associated with early maturation arrest of the B-cell lineage. Mangum et al. have described poorer overall survival associated with this deletion [53]. Disease recurrence occurred as conversion to MRD positivity during maintenance therapy, without overt medullary relapse. He is alive in complete remission with negative MRD. The last case was a boy with dysmorphic features consistent with Goldenhar syndrome. He was diagnosed with B-ALL carrying a t(1;19)(q23;p13). He presented with a hyperdiploid karyotype, and no SNP array or NGS studies were available at diagnosis. He showed a good initial response to treatment. He later relapsed with a complex karyotype characterized by an increased number of chromosomes and the emergence of multiple structural abnormalities in addition to t(1;19), and the disease became refractory to treatment. He ultimately died due to pulmonary aspergillosis in the setting of active disease. Although t(1;19) is considered an intermediate-risk abnormality [54], clinical heterogeneity has been described, with an association with central nervous system relapse and poor outcomes after relapse [55]. In addition, the presence of cytogenetic abnormalities in addition to t(1;19) has been associated with a significantly higher cumulative incidence of relapse in adults [56].

There are three children within the poor cytogenetic risk group who relapsed.

**Figure 7:**
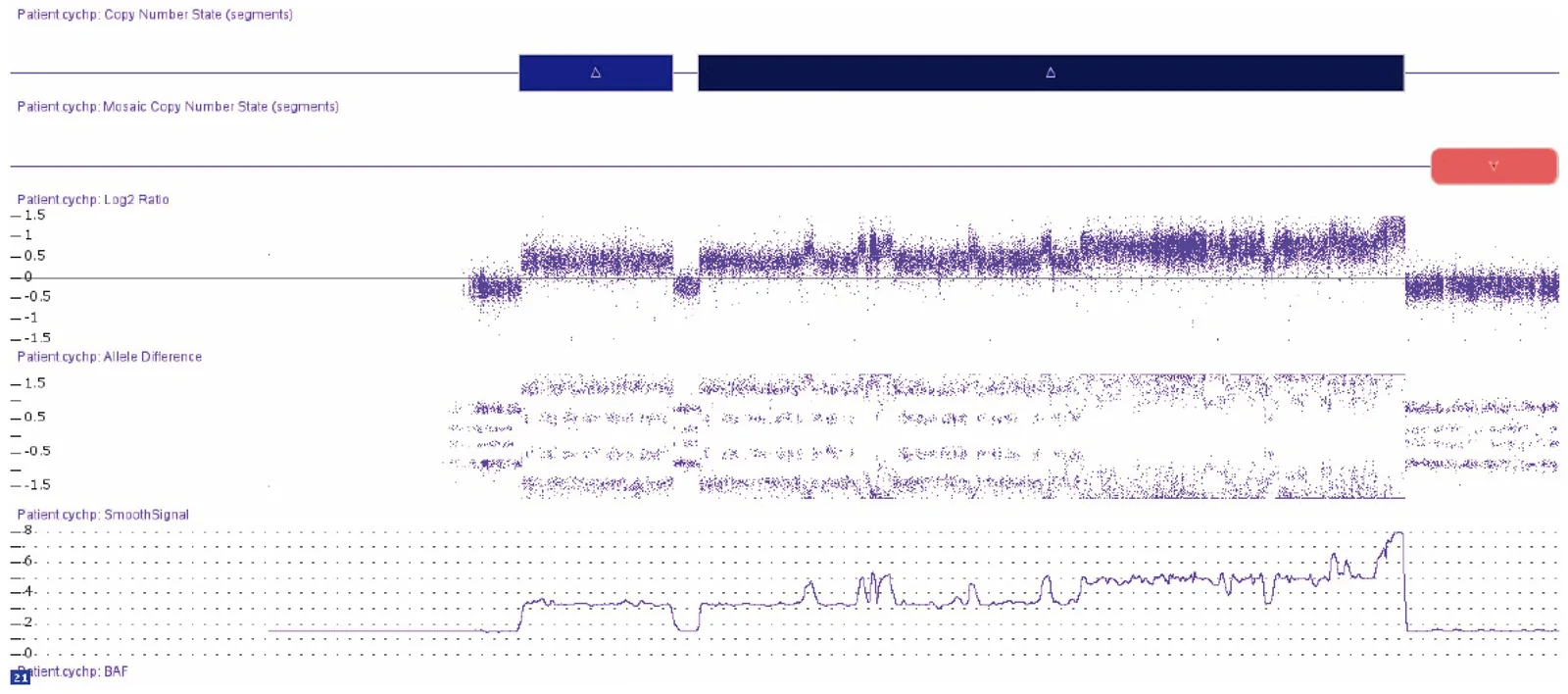
B-ALL with iAMP21. SNP-array analysis demonstrates evidence of iAMP21, as illustrated by alternating copy number changes across the entire chromosome, including amplification encompassing *RUNX1* and terminal copy number loss.

Two children were diagnosed with B-ALL with iAMP21, including *RUNX1* amplification in both cases. The first child presented with extramedullary disease at diagnosis. SNP-array analysis revealed a gain of 6q22 to 6q27 in addition to iAMP21 (Figure 7). The patient showed a good response to treatment, with negative MRD after induction. During follow-up, MRD became positive twice without overt medullary relapse. The patient is currently alive and in complete remission with negative MRD. The second child with iAMP21 also presented a “not good” CNA profile. During disease evolution, an initial poor response to treatment was observed, with >25% blasts on day 15, although MRD became negative after induction. The patient experienced a testicular relapse with bone marrow MRD positivity. The patient is currently alive in complete remission with negative MRD. iAMP21 is characterized by copy number alterations and complex rearrangements within chromosome 21. It is associated with a high risk of relapse when treated with standard protocols, although outcomes have improved significantly with treatment intensification; however, there may also be heterogeneity within this subgroup [57].

**Figure 8:**
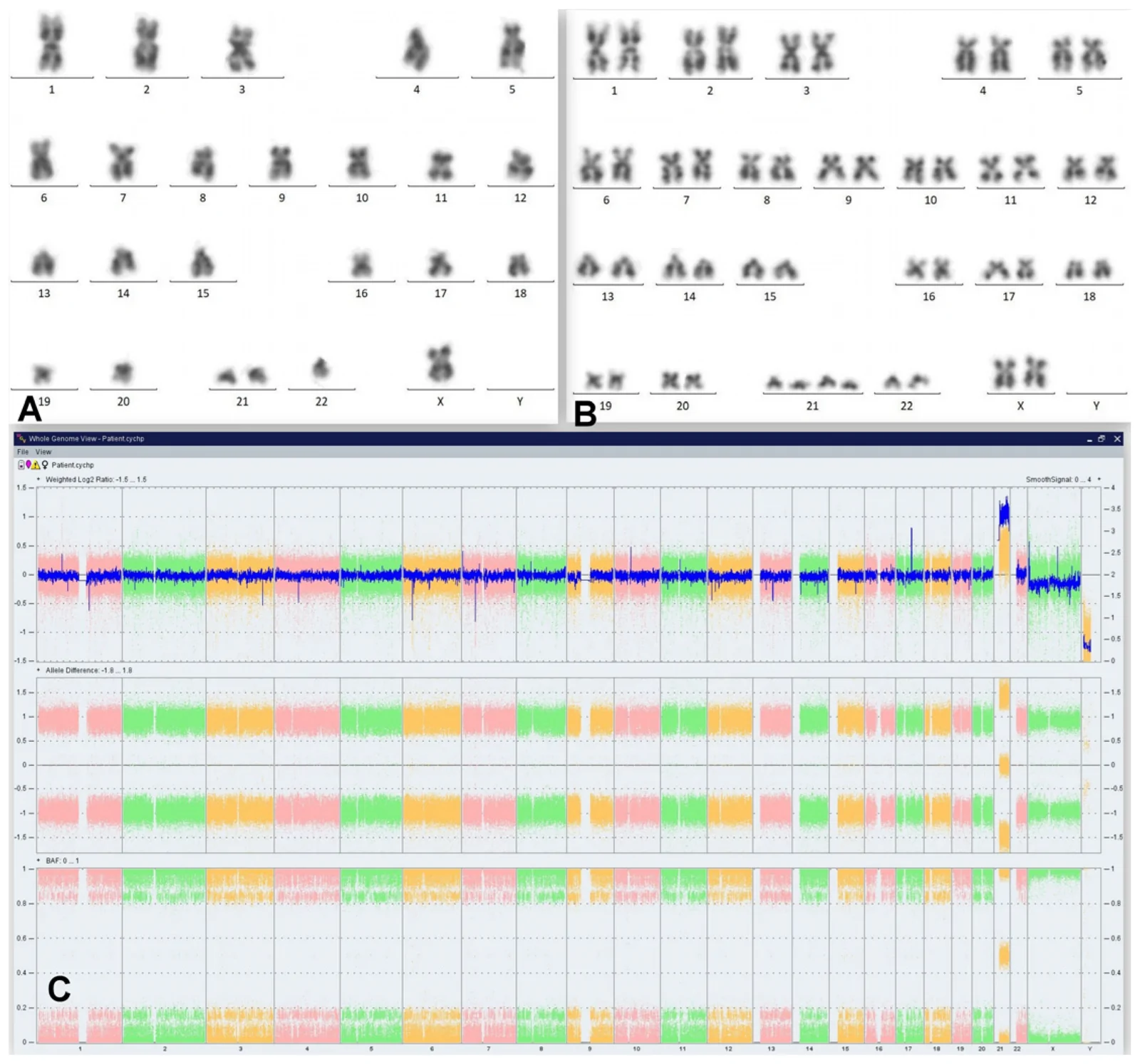
Near-haploid B-ALL. (**A**) Bone marrow karyotype demonstrating a hypodiploid metaphase containing 24 chromosomes. A single copy of each autosome is present, except for chromosome 21, accompanied by loss of the Y chromosome. (**B**) Bone marrow karyotype showing a hyperdiploid metaphase with 48 chromosomes, derived from endoreduplication of the preceding hypodiploid metaphase. (**C**) SNP-array profile. Copy-neutral loss of heterozygosity is observed across all chromosomes except chromosomes 21 and Y, indicating that the disomic state of the remaining chromosomes was achieved through endoreduplication.

The last high-risk genetic finding corresponds to B-ALL with near-haploidy (24 chromosomes). Karyotype analysis identified two abnormal clones (Figure 8): a hypodiploid clone (24 chromosomes) and a hyperdiploid clone (48 chromosomes). SNP-array analysis was essential, demonstrating loss of heterozygosity (LOH) affecting virtually the entire genome, with the exception of chromosomes Y and 21. An additional adverse prognostic factor at diagnosis was patient age (12 years). The disease showed a slow early response, with >25% bone marrow blasts on day 15, although MRD became negative after induction therapy. Near-haploid B-ALL is associated with early relapse, treatment resistance, and poor prognosis, with 5-year survival rates around 20–50% [58]. The patient is currently in relapse following CAR-T cell therapy.

### Limitations

This study has several limitations. The relatively small number of cases and events, particularly within low-frequency subgroups, limits the precision of the estimates and requires cautious interpretation of the associations identified. Another limitation of the study is that the NGS panel comprised only a limited number of genes relevant to B-ALL and was not available for all patients. Consequently, potentially relevant genomic alterations currently recognized as prognostic markers were not systematically assessed. In addition, comprehensive genomic analyses at relapse were available for only a limited number of patients. Although the relapse, when available, retained the same WHO and ICC classification as at diagnosis, paired clonality studies (e.g., immunoglobulin/T-cell receptor rearrangements) were not performed. Therefore, we could not definitively characterize the clonal evolution of the initial leukemia or determine whether relapse arose from the selection of a pre-existing subclone. Furthermore, emerging technologies such as OGM and RNA-seq were not systematically incorporated and may uncover additional genomic alterations that further refine current risk categories.

Finally, other determinants of treatment failure, including acquired mechanisms of drug resistance, were not evaluated and could also influence the risk of relapse.

## 5. Conclusions

Comprehensive genetic characterization of pediatric B-ALL using an integrated diagnostic strategy provides a high diagnostic yield and offers deeper biological insights into the disease at diagnosis. Cytogenetic risk and post-induction measurable residual disease remained the main factors associated with relapse in our cohort, reinforcing their central role in the prognostic stratification of pediatric B-ALL. However, despite the overall favorable outcomes achieved, relapses still occur, predominantly within specific genetic subgroups but occasionally also in patients considered to have favorable-risk disease. These findings suggest that a more comprehensive genomic characterization may contribute to more accurate identification of patients at risk of relapse and, ultimately, help tailor risk-adapted, personalized therapeutic strategies.

## Figures and Tables

**Table 1:** Baseline characteristics and differences according to relapse status.

Characteristic	Overall N = 51 ^1^	Non Relapse N = 39/50 ^1^	Relapse N = 11/50 ^1^	*p*-Value ^2^
**Sex**				0.3
Male	28 (55%)	20 (51%)	8 (73%)	
Female	23 (45%)	19 (49%)	3 (27%)	
**Age (years)**	3 (2, 6)	3 (2, 5)	6 (2, 10)	0.13
**Leukocyte count (×10^9^/L)**	7.70 (3.60, 17.06)	7.20 (3.15, 17.06)	10.70 (4.87, 20.00)	0.2
**Hemoglobin level (g/L)**	8.80 (7.60, 11.10)	8.60 (7.50, 10.70)	10.30 (8.10, 12.70)	0.2
**Platelet count (×10^9^/L)**	58 (37, 111)	61 (37, 111)	58 (22, 116)	0.7
**ICC 2022**				0.022
Hyperdiploid	23 (44.8%)	21 (54%)	2 (18%)	
t(12;21)(p13.2;q22.1)/*ETV6*::*RUNX1*	8 (16.0%)	7 (18%)	0 (0%)	
t(1;19)(q23;p13.3)/*TCF3*::*PBX1*	2 (3.9%)	1 (2.6%)	1 (9.1%)	
*BCR*::*ABL1*-like (*CRLF2*::*P2RY8*)	2 (3.9%)	1 (2.6%)	1 (9.1%)	
iAMP21 ^3^	2 (3.9%)	0 (0%)	2 (18%)	
*PAX5* Altered	2 (3.9%)	1 (2.6%)	1 (9.1%)	
Near haploid	1 (2.0%)	0 (0%)	1 (9.1%)	
Low hypodiploid	1 (2.0%)	1 (2.6%)	0 (0%)	
t(9;22)(q34.1;q11.2)/*BCR*::*ABL1*	1 (2.0%)	1 (2.6%)	0 (0%)	
t(9;11)(p21.3;q23)/*KMT2A*::*MLLT3*	1 (2.0%)	1 (2.6%)	0 (0%)	
*DUX4*-rearrangement	1 (2.0%)	0 (0%)	1 (9.1%)	
NOS ^4^	7 (14%)	5 (13%)	2 (18%)	
**IKAROS^plus^**				0.4
Yes	8 (20%)	5 (16%)	3 (30%)	
No	33 (80%)	26 (84%)	7 (70%)	
**Cytogenetic risk**				0.007
Good risk	29 (57%)	26 (67%)	2 (18%)	
Intermediate risk	17 (33%)	11 (28%)	6 (55%)	
Poor risk	5 (10%)	2 (5%)	3 (27%)	
**CNA ^5^ profile**				0.075
Good	23 (56%)	20 (65%)	3 (30%)	
Not good	18 (44%)	11 (35%)	7 (70%)	
**>25% blast day 15**				<0.001
No	44 (90%)	38 (100%)	6 (55%)	
Yes	5 (10%)	0 (0%)	5 (45%)	
**MRD ^6^ day 33**				0.078
Positive	19 (38%)	12 (31%)	7 (64%)	
Negative	31 (62%)	27 (69%)	4 (36%)	
**MRD ^6^ end of induction**				0.017
Positive	6 (12%)	2 (5%)	4 (36%)	
Negative	44 (88%)	37 (95%)	7 (64%)	
**Death**				0.001
Yes	4 (8.0%)	0 (0%)	4 (36%)	
No	46 (92%)	39 (100%)	7 (64%)	

^1^ n (%); Median (Q1, Q3). ^2^ Fisher’s exact test; Pearson’s Chi-squared test; Wilcoxon rank sum test. ^3^ Intrachromosomal amplification of chromosome 21. ^4^ Not otherwise specified. ^5^ Copy number alteration. ^6^ Measurable residual disease.

**Table 2:** Firth-penalized Cox univariate analysis.

Variable	N (Events)	Contrast	HR ^1^	95% CI ^2^	*p* -Value
Cytogenetic risk	50 (11)	Intermediate risk vs. good risk	4.09	0.86–19.4	0.044
Cytogenetic risk	50 (11)	Poor risk vs. good risk	7.21	1.27–40.9	0.020
End of induction MRD ^3^	50 (11)	Positive vs. negative	10.2	2.78–37.4	0.001
>25% blasts at day 15	49 (11)	Yes vs. No	13.4	3.53–50.8	<0.001
MRD ^3^ day 33	50 (11)	Positive vs. negative	2.32	0.68–7.87	0.2
CAN ^4^ profile	41 (10)	Good profile vs. Not good profile	2.94	0.77–11.3	0.085

^1^ Hazard ratio. ^2^ 95% confidence intervals. ^3^ Measurable residual disease. ^4^ Copy number alteration.

**Table 3:** Firth-penalized Cox Multivariable analysis: main model.

Model	Variable	Contrast	HR ^1^	95% CI ^2^	*p* -Value
**Main model**					
	Cytogenetic risk	Intermediate risk vs. good risk	2.56	0.50–13.1	0.300
	Cytogenetic risk	Poor risk vs. good risk	9.31	1.55–56.1	0.010
	End of induction MRD ^3^	Positive vs. negative	10.9	2.39–49.9	0.002

^1^ Hazard ratio. ^2^ 95% confidence intervals. ^3^ Measurable residual disease.

**Table 4:** Description of relapses.

GS ^4^ (Months)	Death/ Cause of Death	Treatment After Relapse	RFS ^3^ (Months)	End of Induction MRD ^2^	Treatment	NGS (Diagnosis//Relapse)	CAN ^1^ (Diagnosis//Relapse)	Cariotyping and FISH (Diagnosis//Relapse)	**Extramedular Disease**	**Leucocyte Count**	**Age/Sex**	**ICC 2022**
**CYTOGENETIC GOOD RISK**												
45	No	CAR-T	19	+	A* High Risk + allo-HSCT ^7^	*KRAS*, *IKZF1*/ KRAS, IKZF1	*CDKN2A/B* LOH 12 y 13 *ETV6*//NA ^6^	54, XY, +X,+4,+6,+8,+14,+17,+21,+21//NA ^6^	Yes (NCS ^5^)	10.07	8/ Male	**(1) Hyperdiploid**
50	No	Chemoterapic treatment + allo-HSCT ^7^	26	-	A* Standard risk	NA ^6^// *FLT3*, *KRAS*	Negative // *PAX5*	54-57,XX,+X,+4,+5,+6,+8,+14,+14,+17,+21,+21,+21[cp15]/46,XX[5] //55,XX, +X,+4,+6,+8,+14,+14,+17,+21+21	No	3.76	3/Female	**(2) Hyperdiploid**
**CYTOGENETIC INTERMEDIATE RISK**												
30	Yes// relapse	Inotuzumab, CAR-T	20	-	A* Intermediate risk	IKZF1// NA ^6^	*CDKN2A/B*, *ERG, TBL1XR1*// *CDKN2A/B*, *ERG*, *TBL1XR1*, LOH 19p13.3-p13.2	47,XY,+21[2]/46,XY[8] //FISH negative	Yes (maxillary involvement)	7.5	12/ Male	**(3) *DUX4-* rearrangement**
26	Yes//pulmonary hemorrhage	CAR-T, Inotuzumab	15	+	A* High risk + allo-HSCT ^5^	Negative //NA ^6^	*CDKN2A*/2B, *PAX5*, *TP53* Near-triploidy /Near-tetraploidy, i(17)(q10)	61–78,XXX,inc[cp6]/91-107,XXXX,inc[cp3]	No	13.9	10/Female	**(4) NOS ^8^**
42	No	Blinatumumab, Innotuzumab + allo-HSCT ^7^, CAR-T	9	+	B* Good risk	*KRAS*// NA ^6^	*IKZF1*, *CDKN2A/2B*, *PAX5* *PAX5*::*NOL4L*// NA^6^	45,XX,del(6)(q16.1q22.33),dic(9;20)(p13.2;q11.21) [10]// NA ^6^	No	122.45	0.9/Female	**(5) *PAX5* altered**
37	Yes// septic shock	Chemoterapic treatment (SEHOP/PETHEMA 2015 relapse protocol)	35	+	A* Intermediate risk	NA ^6^// NA ^6^	*CRLF2*::*P2RY8* *IKZF1*, *CDKN2A/2B*, *PAR1*, *PAX5*// NA ^6^	45,XY,-7,add(9)(p13.2),del(11)(p11.2),add(12)(q22)[12]/46,XY[8] FISH negative // NA ^6^	No	28.6	1/ Male	**(6) *BCR*::*ABL*-like (*CRLF2*::*P2RY8*)**
39	No	Chemoterapic treatment + allo-HSCT ^7^	14	-	A* Intermediate risk	Negative// Negative	22q11.22 (*VPREB1*). No CNA ^1^// NA ^6^	No growth/FISH negative// NA ^6^	No	10.5	6/ Male	**(7) NOS ^8^**
26	Yes// invasive pulmonary aspergillosis	Chemoterapic treatment, CAR-T + allo-HSCT ^7^	11	-	A* Intermediate risk	NA ^6^// Negative	NA ^6^// NA ^6^	50,XY,t(1;19)(q23;p13.3),+4,+5,+8,+?[6]/46,XY [1]. FISH: *TCF3*/*PBX1*// ~57,XX.+1,t(1;19)(q23;p13.3),add(5)(q32),+6,+8,+8,+11, +14,+14,+21,+22,+mar1,+mar2[20]	No	12.4	2/ Male	**(8) t(1;19)(q23;p13.3)** **/*TCF3*::*PBX1***
**CYTOGENETIC HIGH RISK**												
32	No	Blinatumumab, Inotuzumab + allo-HSCT ^7^	20	-	A* Intermediate tisk	NA ^6^// Negative	iAMP21 ^7^// NA ^6^	No growth/FISH: iAMP21 ^9^// NA ^6^	Yes (lymph node and soft tissue involvement)	4.26	7/ Male	**(9) iAMP21 ^9^**
29	No	CAR-T + RT	24	-	A* High risk + allo-HSCT ^7^	NA ^6^//NA ^6^	iAMP21 ^9^, *IKZF1*, *ETV6*	No growth, FISH negative// NA ^6^	No	20	6/ Male	**(10) iAMP21 ^9^**
24	No	CAR-T, chemoterapic treatment	20	-	A* High risk	Negative// Negative	*IKZF1*, *CDKN2A/2B*, *BTG1*, *EBF1*, *PAX5*, *ETV6*, *RB1*, *TP53*, *IKZF1*^plus^// NA ^6^	24<n>,X,-Y,+21[10]/48,idemx2 [8]/46,XY[2]// 24<n>,X,-Y,+21[1]/48,idemx2 [4]/46,XY[15]	No	4.9	12/ Male	**(11) Near haploid**

^1^ Copy number alteration. ^2^ Measurable residual disease. ^3^ Relapse free survival. ^4^ Global survival. ^5^ Central Nervous System. ^6^ Not available. ^7^ Allogeneic transplant. ^8^ Not otherwise specified. ^9^ Intrachromosomal amplification of chromosome 21. A* SEHOP-PETHEMA 2013. B* Interfant-06.

## Data Availability

The raw data supporting the conclusions of this article will be made available by the authors on request.

## Supplementary Materials

The following supporting information can be downloaded at: https://www.mdpi.com/article/10.3390/cancers18162633/s1, Table S1: Univariable Firth-penalized logistic regression; Table S2: Firth-penalized Cox Multivariant analysis: sensitivity analysis.
