# Supplementary material for: Pediatric B-Cell Acute Lymphoblastic Leukemia: Comprehensive Genomic Characterization Including SNP-Array and Analysis of Relapse Risk
Source: Cancers (Basel). 2026 Aug 14;18(16):2633. doi: 10.3390/cancers18162633 (PMC13511422; doi:10.3390/cancers18162633)
Supplement: Supplementary file 1 [file cancers-18-02633-s001.zip › cancers-4441403-supplementary.pdf]

Supplementary Table S1. Univariable Firth-penalized logistic regression

In univariable Firth-penalized logistic regression models for relapse, cytogenetic risk was significantly associated with relapse (N=50), showing a stepwise increase compared with the favorable group: Intermediate OR 5.99 (95% CI 1.30–36.8; p = 0.021) and poor risk OR 14.8 (95% CI 1.99–144; p = 0.009). End-of-induction MRD positivity was associated with higher odds of relapse compared with MRD negativity (N=50; OR 9.00; 95% CI 1.67–60.4; p = 0.011). The presence of >25% blasts on day 15 showed a very strong association with relapse (N=49; OR 65.2; 95% CI 6.23–8,952; p < 0.001), although the confidence intervals were wide due to sparse data. Day 33 MRD showed a non-significant trend toward higher relapse (N=50; OR 3.67; 95% CI 0.98–15.2; p = 0.053). In the subset with available CNA profile data (N=41), a non-significant trend toward higher relapse was observed for "not good" versus "good" (OR 3.82; 95% CI 0.93–18.5; p = 0.062). Overall, wide confidence intervals reflect the limited precision expected with a small number of events and sparse categories.

Characteristic	N	Exp (beta)	95% CI[1]	p-value
**Cytogenetic risk**	50			
Good risk		-	-	
Intermediate risk		5.99	1.30, 36.8	0.021
Poor risk		14.8	1.99, 144	0.009
**End of induction MRD[2]**	50			
Positive		9.00	1.67, 60.4	0.011
Negative		~~9.00~~ -	~~1.67, 60.4~~ -	
**>25% blasts at day 15**	49			
No		-	-	
Yes		65.2	6.23, 8,952	<0.001
**MRD[2] day 33**	50			
Negative		-	-	
Positive		3.67	0.98, 15.2	0.053
**CNA[3] profile**	41			
Good risk		-	-	
Not good risk		3.82	0.93, 18.5	0.062

1 95% confidence intervals.
2 Measurable residual disease.
3 Copy number alteration.

Supplementary Table S2. Firth-penalized Cox Multivariant analysis: sensitivity analysis

Model	Variable	Contrast	HR[1]	95% CI[2]	p-value
**Sensitivity analysis: >25% blasts at day 15**					
	Cytogenetic risk	Intermediate risk vs good risk	3.62	0.76–17.3	0.068
	Cytogenetic risk	Poor risk vs good risk	1.09	0.12–9.53	>0.9
	>25% blasts at day 15	Yes vs No	18.8	3.11–114	0.001
**Sensitivity analysis: MRD[3] day 33**					
	Cytogenetic risk	Intermediate risk vs good risk	4.65	0.97–22.3	0.028
	Cytogenetic risk	Poor risk vs good risk	7.06	1.23–40.5	0.022
	MRD day 33	Positive vs negative	2.60	0.75–8.97	0.11

1 Hazard ratio.
2 95% confidence intervals.
3 Measurable residual disease.
